# Case report: Adult with bipolar disorder and autism treated with ketamine assisted psychotherapy

**DOI:** 10.3389/fpsyt.2024.1322679

**Published:** 2024-02-20

**Authors:** Christopher P. Harris, Becky Jones, Kathryn Walker, Meredith S. Berry

**Affiliations:** ^1^Revitalist Lifestyle and Wellness Ltd., Knoxville, TN, United States; ^2^Department of Health Education and Behavior, University of Florida, Gainesville, FL, United States; ^3^Department of Psychology, University of Florida, Gainesville, FL, United States

**Keywords:** ketamine, ketamine assisted psychotherapy, Autism Spectrum Disorder, bipolar disorder, psychopharmacology, case report

## Abstract

**Background:**

Evidence has increased in recent years regarding the potential for ketamine to serve as a novel treatment option for a range of conditions, particularly depression (unipolar and bipolar). However, research regarding ketamine as a potential therapeutic for Autism Spectrum Disorder (ASD) is lacking, despite high overlap with bipolar depression and theoretical foundations for its use.

**Case presentation:**

A 29-year-old man with bipolar disorder and Autism Spectrum Disorder, type 2 diabetes, presented with mood swings and suicidal thoughts, and anger outbursts occurring daily. The patient was referred by a psychiatrist due to irritability and outbursts during the previous 5 months. These outbursts were unable to be controlled by the medications prescribed, included yelling and screaming, and the patient was unable to speak with the psychiatrist. The patient underwent ketamine assisted psychotherapy with 6 initial IV infusions of ketamine over a 1 month period followed by 2 booster IV ketamine infusions. Following ketamine treatment, dramatic reductions in outbursts were observed as well as reductions in anxiety, suicidality, and depression scores.

**Conclusion:**

This case study adds to the scant literature regarding ketamine treatment for individuals with bipolar disorder and ASD. We did not find ASD to be a contraindication for IV ketamine and ketamine assisted psychotherapy. Reductions in anger outbursts, anxiety, suicidality, and depression suggest ketamine treatment might be tailored to individuals with bipolar disorder and ASD, and additional systematized research is warranted. Although potential mechanisms of action are not clear, these data add to the discussion regarding clinical practice considerations and the potential for ketamine to improve quality of life and associated metrics.

## Introduction

Clinical evidence of an association between Bipolar Disorder (BD) and Autism Spectrum Disorder (ASD) is well documented (1–4) with both conditions possibly sharing the same genes (3). Comorbid conditions with ASD, including BD, can negatively impact quality of life and significantly increase difficulties in adaptive responses and daily activities, and can increase problems such as aggressiveness, irritability, and social isolation (5–9). Bipolar Disorder in particular can affect up to 8% of individuals with autism (6), may exacerbate ASD associated impairment of social and cognitive functioning, and present diagnostic and treatment barriers (6–9). In order to better treat comorbid ASD and BD, innovative therapeutics are needed to target aspects of both conditions.

Emerging evidence points toward promising results in the treatment of BD with ketamine, a non-competitive N-methyl-D-aspartate receptor antagonist. Recent publications and reviews of the literature demonstrate an abundance of evidence supporting ketamine as a robust, rapid, and transient antidepressant with anti-suicidal effects in unipolar and bipolar depression (10–16). The use of ketamine with ASD, however, is not well researched – although there appear to be theoretical applications for its use (17–21).

This report presents the case of a 29-year-old male with autism and bipolar disorder being treated with IV Ketamine. To our knowledge, a case as particular as this has not been reported in the literature with the treatment regimen of 6 initial IV ketamine infusions. Other research, however, has shown that a single participant study conducted with an adult with ASD found promising results with the patient’s mood (19). Another case report (20) detailed a 15-year-old male with autism, bipolar, and obsessive-compulsive disorder who received ketamine as part of a dental procedure at the Special Needs Dentistry Center at the Rose F. Kennedy Center. Immediately upon recovery from the procedure, the child was speaking in full sentences and making eye contact. These were remarkable differences from the patient’s typical behavior; however, this change only lasted about 36 h and then quickly waned. The study cautioned that this single case study should not be seen as evidence to recommend the use of ketamine for the treatment of autism at present. Another double-blind study among adolescents with autism found that while intranasal ketamine was well tolerated, no significant improvements were observed using the Aberrant Behavior Checklist Social Withdraw (18).

We agree with previous authors and acknowledge the present report is a single case report of an individual with autism and bipolar disorder, seeking care for bipolar episodes. This case report should not be overgeneralized, but initial data warrant additional research. Here we present the case of an adult male with autism and bipolar disorder treated with ketamine, which results in significant improvements in bipolar episodes with some alleviation of autistic symptoms. He was treated at the Revitalist Clinic (established in 2018) in 2021 and is still currently receiving care.

## Case description

### Patient information

A white male, weighing 185 lbs, presented with bipolar disorder, Autism Spectrum Disorder, type 2 diabetes, complaining of mood swings with suicidal thoughts and anger outbursts. These outbursts occurred daily since the summer of 2021 and for 4–5 months prior to seeking ketamine treatment, prompting his referral to Revitalist by his primary psychiatrist.

His mother reports a “long labor” and a week-long stay in the Neonatal Intensive Care Unit, due to breathing problems. As early as a newborn he exhibited “rocking” behaviors while in a crib. At age 2, he was diagnosed with Autism from a university developmental and genetics center. He began receiving speech lessons at 2 ½ years old. At age 4 he was able to put 2 words together and speech and sensory integration lessons continued until he was 11.

As a child, the patient struggled with attention and concentration. He had repetitive and ruminating thoughts and behaviors. His social skills were poor, and his speech was slow. His vocabulary, however, was excellent. He struggled with gross motor skills but was able to develop unremarkable fine motor skills. He had visual processing issues, for example, he would see things upside down in eye tests. The mother reported auditory processing delays as well, stating that he would often not respond verbally to things said in a conversation but later would come back and report that he heard and understood the conversation.

The patient’s medical history includes an adult-onset of type 2 diabetes mellitus diagnosed at 19 years old. Bipolar disorder was diagnosed at age 18 within 6 months of receiving care from his psychiatrist. The patient is currently under the care of a psychiatrist, maintains a job with developmental disability support at a grocery store, and graduated from high school.

The patient has been treated for bipolar disorder for 12 years. The patient’s mother and psychiatrist listed multiple prescribed medications that ultimately left the patient over sedated and had no therapeutic effect or therapeutic effect decreased over time. Currently, the patient is being treated with a comprehensive medication regimen, which includes lithium carbonate, clonazepam, lumateperone, choline, doxycycline, iloperidone, fluticasone propionate, inositol, lamotrigine, Lantus Solostar U-100 insulin, magnesium, Metformin, a multivitamin, and vitamins B6, B12, C, and E.

Episodes typically consisted of the patient being triggered by something that would make him angry. He would then express urges to hit his parents, cursing, and making grimacing faces. He would then run to his room, hit walls, express the will to not live, slam doors, and call names. The name-calling, the mother described, were names of vocations that the patient felt were insults, but in reality, were not insults. These episodes grew more frequent during the summer of 2021 and began occurring daily.

### Family history

The patient was raised by his biological parents. Both mother and father report histories of depression and currently being in treatment. The patient has a younger sister who the patient reports having a healthy relationship. The sister reports no mental or developmental disorders.

### Clinical findings

Patient presented as well-groomed, healthy-appearing, well-nourished, and well-developed. He was oriented to situation, time, place, and person. He made limited eye contact. His speech was fluent, clear but also loud and drawn out. He appeared to have a good vocabulary. At times his speech was childlike and would switch to deeper and age-appropriate. His judgment and thought processes were intact and logical. He appeared to have a hand tremor. There was no evidence of hallucinations and delusions. The patient reported suicidal thoughts but denied a plan or intent to act. His affect was flat and depressed. Characteristics of ASD were observed (difficulties in maintaining eye contact, language peculiarities, social interaction, specific interests, and rituals).

### Diagnostic assessment and therapeutic intervention

This case was unique given the unknown ketamine response given the overlap in symptoms of ASD and bipolar disorder. Following an initial consultation (details below), a ketamine infusion regimen in combination with psychotherapy was recommended by the treatment team. Table 1 depicts the infusion schedule and Table 2 shows the pre and post ketamine treatment scores on outcome measures (details below).

**Table 1:** Denotes the timeline of ketamine infusions.

Consultation/baseline	Infusion	Infusion	Infusion	Infusion	Infusion	Infusion	Booster	Booster
Day 1	Day 3	Day 8	Day 15	Day 17	Day 22	Day 24	Day 69	Day 93

**Table 2:** Displays participant scores for all scales administered (GAD-7; CSSRS; PHQ-9; PCL-5; ACE) before ketamine treatment (baseline) and following Infusion 6, and boosters 1 and 2.

Scale*	GAD-7	CSSR-S	PHQ-9	PCL-5	ACE
Baseline**	21	2	12	52	1
Infusion 6	12	0	8	26	n/a
Booster 1	18	1	10	35	n/a
Booster 2	11	0	6	21	n/a

*Lower is better for all scales, ** Infusions 2–5 omitted. See text for details.

A psychiatric nurse practitioner performed the initial consultation. Chief complaints were frequency and severity of bipolar episodes and presence of suicidal ideation. At baseline, the patient scored actionable on all scales and the highest possible score on the anxiety scale, indicating severe anxiety (see Table 2). Prior to and following infusions, the patient completed the General Anxiety Disorder-7 (GAD-7), Columbia-Suicide Severity Rating Scale (C-SSRS), Patient Health Questionnaire (PHQ-9), Posttraumatic Stress Disorder Checklist (PCL-5), and the Adverse Childhood Experiences (ACE) scales.

Given the patient’s suicidal ideation, high anxiety, and outbursts, he was recommended 6 initial ketamine infusions, each administered as a 40-min IV infusion without a push, in addition to psychotherapy, with a minimum interval of 48 h between sessions (21). The protocol for intravenous ketamine infusion typically involves the establishment of an IV line for the administration of ketamine. Dosage is calculated based on body weight, and administered incrementally to monitor the patient’s physiological and psychological responses. The duration of each infusion session generally spans 40–60 min. Throughout this period, vital parameters including heart rate, blood pressure, and oxygen saturation, are rigorously monitored to ensure patient safety. The infusion environment is deliberately structured to be tranquil and supportive, with reduced external stimuli, thereby facilitating a conducive setting for the patient.

Following the infusion, patients undergo a period of observation to manage and assess any immediate side effects, such as disorientation or dizziness. Concurrently, the therapeutic efficacy of the ketamine treatments is evaluated in synergy with the ongoing psychotherapeutic interventions. Adjustments to the ketamine treatment protocol are made contingent upon the patient’s evolving response and the overarching therapeutic objectives.

Booster infusions following the initial infusions were given as needed (see Table 1). For the first infusion, a standard of 40 mg intravenous ketamine dose was titrated by 10 mg for each infusion following. The 6th infusion ended at 90 mg of ketamine and was well tolerated. Further booster infusions were 90 mg and 110 mg, respectively. Blood pressure, heart rate, and oxygen saturation were monitored in consistent intervals. The proportion of ketamine given and the levels of titration were all standards of care and procedure, and did not deviate because of his diagnosis of ASD.

The patient was assigned a clinician holding a Master’s in Social Work, specializing in mental wellness coaching. This professional provided ketamine-assisted psychotherapy (KAP) along with subsequent integration sessions, supplementing the ketamine treatment. Ketamine assisted psychotherapy (KAP) is a therapeutic approach that combines the administration of ketamine with psychotherapeutic techniques. This method provides safety and structure during ketamine infusions, facilitating a therapeutic environment. KAP aims to enhance the psychological insights and therapeutic effects of ketamine by integrating it with psychotherapeutic support, including guided imagery, and reflective discussion. The approach allows for the exploration and processing of emotional and cognitive experiences that arise during the ketamine-induced altered state of consciousness, promoting psychological healing and personal growth.

Integration sessions occur after a person has experienced a non-ordinary state of consciousness from a psychedelic induced therapeutic environment. Integration allows the synthesis of mind and body and helps the person create meaning from the non-ordinary experience. In addition to the integration of the ketamine experience, mental wellness coaching also followed the 6th infusion. Mental wellness coaching consists of repetitive, solution-focused, task-oriented homework and reality-based coaching using Cognitive Behavioral Therapy (CBT). These additional wellness coaching sessions were deemed an appropriate fit given his goals of “being less autistic” and “having a successful life.” Booster infusions were completed simultaneously with the twice-a-week coaching sessions at 3.5-week intervals.

### Follow-up and outcomes

**Figure 1:**
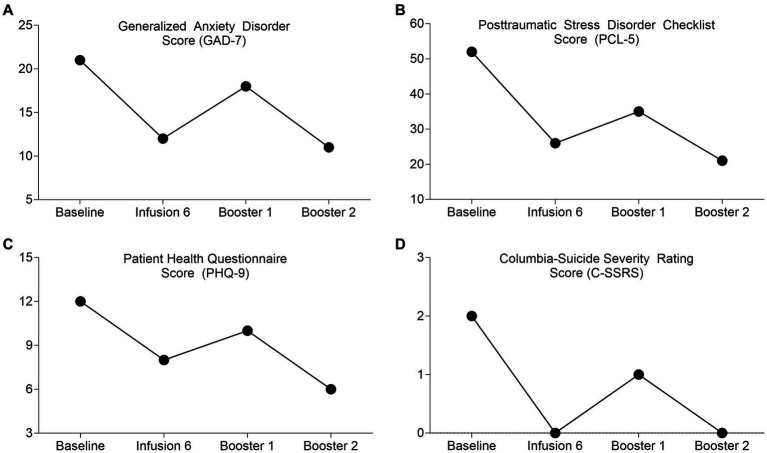
Displays the responses of the patient prior to (Baseline), during (Infusion 6 and Booster 1), and following the final dose (Booster 2) of the ketamine assisted psychotherapy treatment for the scales administered. For all scales, lower scores represent less severe symptomology. Panel **(A)** displays scores for the Generalized Anxiety Disorder (GAD-7), Panel **(B)** scores for the Posttraumatic Stress Disorder Checklist Score (PCL-5), Panel **(C)** scores for the Patient Health Questionnaire (PHQ-9), and Panel **(D)** scores for the Columbia-Suicide Severity Rating Scale (C-SSRS).

Meaningful reductions in anxiety (GAD-7), suicidality (CSSR-S), depression (PHQ-9), and Posttraumatic Stress (PCL-5) were observed following ketamine treatment (Table 2). Pre and post infusion results for these outcome measures are presented in Table 2 and Figure 1. No adverse events were observed by the clinical team or reported by the patient or caregivers.

### Patient perspective

The patient is under the care of his parents, who have power of attorney. The patient and parents consented to be interviewed. Prior to the ketamine treatment, the patient’s mother noted that the patient had experienced daily episodes since the summer of 2021, characterized typically by bouts of anger and suicidal outbursts. She reports that his medication was not working and that his lithium and clonazepam were not working to quell the episodes. The mother reports that “ketamine has opened a door for therapy for him and allowed him to think about things differently.” The mother reported that he has had an episode about once every 2 weeks now. She reported that transitions were difficult for him. She stated they chose to take a family vacation and he had one episode when they were packing up to leave. The mother reports that he seems to have insight into his anxious states and will verbally report anxiety and ask for medication, which are both improvements.

The mother reported his obsessive thoughts and behaviors have also improved and stated positively that his “thoughts have slowed since the ketamine.” The mother stated, “things have been a lot more peaceful around here” and that “he looks forward to the infusions and therapy.” The mother reported that “he is learning skills in therapy and understands why he does what he does.” The mother reported no significant side effects or issues with treatment. The patient appeared calm in the interview and reported that ketamine helped him feel calm. The patient reported he was glad he did it and that he liked therapy because it “helps me feel better.” All parties reported feeling happy and satisfied with the treatment that the patient received.

### Referring provider perspective

The patient was referred by a professional concurrently serving as a psychiatrist and an assistant professor, with a career spanning over 32 years in the field. The MD referred to the patient as being in the moderate range for ASD and severe Bipolar Disorder. The MD reported the patient struggled with self-care but was verbal and had no signs of neurological deficits. The MD reports treating the patient with several medications for mood which ultimately were ineffective or left the patient over sedated. The patient was referred due to irritability and outbursts uncontrolled by medication. The MD was interviewed following the patient’s 7th session. The MD reported that during his session with the patient before ketamine, the patient was yelling and screaming and was unable to speak with the physician. Following the infusions, the MD reports the patient was calm, laid back, and was able to converse.

## Discussion

This case report is the only known report in the literature of a patient with ASD and Bipolar disorder who received a series of 6 initial infusions of IV ketamine, followed by booster infusions. The young man presented with uncontrolled anger episodes, high anxiety, and suicidality, with comorbid ASD and Bipolar Disorder at the Knoxville Revitalist Clinic. Anger outbursts, symptoms of anxiety, depression and suicidality were all substantially reduced following ketamine treatment. The patient’s unique comorbidity enabled him to receive therapeutic care that may inform the standard of care for future patients with these conditions.

The strength of this report is the multiple interviews, details of multi-disciplinary care, and the thorough follow-up. The limitations were the lack of ASD scales and assessments, which leave improvements in his ASD symptoms up to the subjective reports in the interviews. The patient’s symptoms support the diagnosis of ASD and Bipolar Disorder. Bipolar Disorder co-occurs with an ASD diagnosis much more than without (6–9). We however caution that expertise in ASD and mood disorders is vital in establishing a diagnosis. Given the overlap in symptoms of ASD and Bipolar Disorder, an accurate diagnosis is key to a reliable treatment plan. A particular strength of this case report is that the patient was referred to the clinic by a psychiatrist who had seen the patient for well over a decade and has been in practice for 32 years, and thus was able to provide an accurate diagnosis, detailed assessment, and note improvements after ketamine treatment.

The patient presented at admission with suicidal ideation, which is high among those with ASD and mood disorders (22, 23). Protective factors with ASD and suicidality are not well documented in the literature, although social support has been reported in some cases (24). The patient did have reports of social interpersonal issues being a topic during the treatment. We also hypothesize based on research outside of ASD, that warm, attentive and engaged parents may serve as a protective factor, along with engagement in treatment. The patient’s treatment compliance was high and his results were positive. The patient successfully developed a rapport with the assigned therapist. Continued engagement in coaching sessions with this therapist beyond the sixth infusion was observed to yield positive outcomes. The patient’s desire to work on “having a successful life” also shows hope for the future. His continued denial of suicidal ideation after his 3rd infusion until his most recent booster, 3 months since his first infusion, is another positive sign.

In the previous case report discussed, the patient with ASD responded initially to a single infusion of ketamine but results faded shortly after. Other studies found adolescents with ASD did not respond to intranasal ketamine with multiple administrations (18) with one single participant study showing improved mood from intranasal ketamine (19). In terms of depression, a recently published study in Sweden showed that ketamine’s effect on depression was not short-acting as previous research had found (13). Rather 50% of participants reported being in remission at about 150 days with 30% still reporting remission after 6 infusions at 12 months. Although more research is needed, ketamine has been shown to be both beneficial for those suffering from bipolar depression as well as well tolerated (10, 16). With the unique links between ASD and bipolar disorder, we believe that further research with ketamine is warranted with this specific population.

It is important to note that while the patient did not receive any assessments for his ASD, it did seem that, per the interviews, his ASD symptoms showed improvement. These results are not however reported on any scale or assessment, and so cannot be distinguished objectively from progress on his mood and anxiety. It is possible that the relief he experienced from his depressive symptoms allowed him to focus on treating his ASD symptoms more effectively rather than the treatment itself relieving him of his ASD symptoms. It is also possible that the patient’s ASD symptoms may have been improved by the KAP he received during the ketamine infusions. Given the close connection between ASD and bipolar disorder, it is also plausible that the ketamine infusions may have alleviated some of his ASD symptoms, as eye contact, speech, social interaction, and insight into his emotional state showed improvement following the ketamine infusions. These results also align with another report that some ASD symptoms improved briefly with a single dose of ketamine (20). Taken together, prospectively designed, systematized research with larger sample sizes appears warranted.

The patient’s mother had reported that the patient had not previously experienced outpatient psychotherapy. The inclusion of KAP during the infusions seemed to benefit the patient. Post booster infusions coupled with therapy, with an emphasis on coaching, also worked well with this patient. The mother had reported a fear that outpatient counselors would not be able to understand the patient and as a result, that therapy would ultimately fail, which led to her decision to not enroll him in counseling. The success of therapy, in this case, may be related to ketamine administration, as ketamine has been shown to produce euphoric effects (15). It is possible that such euphoric or related effects of ketamine facilitated his ability to bond and build rapport with his therapist, setting the foundation for enhanced therapy-related outcomes. A case study in 2021 theorized that ketamine and psychotherapy may act synergistically, with therapy augmenting the response to treatment and repeated sessions contributing to the durability of the effect (21). It may also be that the administration of KAP, which frequently renders the patient vulnerable and open to influence, could notably promote the formation of a therapeutic alliance between the patient and therapist. This effect is likely enhanced by the therapist’s active presence and support during this pivotal stage, potentially bolstering the patient’s perception of safety and fostering a deeper trust in the therapeutic relationship. Studying the synergy of ketamine and therapy, as well as disentangling the effects of both components on ASD and bipolar symptoms could represent a beneficial area of future research.

Importantly, we did not observe any evidence in this case report to suggest that an ASD diagnosis should exclude a patient from ketamine treatment. When making decisions on treatment for ASD it is important to note that prior treatments for ASD that include infusions with secretin were found to only have transient effects (25). No suggestion of generalizability for the effectiveness of ketamine with ASD is being made or given in this case report.

Access to ketamine infusions can be difficult as IV ketamine is considered an off-label treatment for depression and therefore insurance may not cover the full cost of treatment. Esketamine, an enantiomer of ketamine, has been developed under the brand name Spravato^™^ in intranasal form, and is approved by the Food and Drug Administration (FDA) for the treatment of depression. However, a single study discussed previously showed no efficacy with the relief of ASD symptoms (18). A separate case study did however report improvement with depressive symptoms and duration of eye fixation with a patient with ASD (19).

## Conclusion

Persons with ASD and bipolar disorder require expertise in diagnosis and treatment. Extensive care and assessment should be given before a diagnosis is made as the process can take longer due to extended and repeated assessments. We did not find ASD to be a contraindication for IV ketamine and KAP. The success of this case is a hopeful step toward a call for more research and further examination of ketamine as a treatment for autism. Client-centered care and collaboration with the parents and referring physician, are recommended and were keys to the success of the treatment. The size of the clinic in proportion to the community it serves also benefited the client in that individualized and close attention was given with the addition of thorough follow-up care.

## Data availability statement

The original contributions presented in the study are included in the article/supplementary material, further inquiries can be directed to the corresponding authors.

## Ethics statement

Ethical review and approval was not required for the study on a human participant in accordance with the local legislation and institutional requirements. Written informed consent was obtained from the individual for the publication of any potentially identifiable images or data included in this article. The authors declare that information from this case study was collected in accordance with the standard of care.

## Author contributions

CH: Conceptualization, Data curation, Investigation, Methodology, Writing – original draft. BJ: Conceptualization, Data curation, Project administration, Writing – review & editing. KW: Conceptualization, Investigation, Project administration, Resources, Supervision, Writing – review & editing, Data curation. MB: Visualization, Writing – review & editing.
